# Application of *n*-of-1 treatment trials in schizophrenia: systematic review

**DOI:** 10.1192/bjp.2018.71

**Published:** 2018-07

**Authors:** Katie F. M. Marwick, Anna J. Stevenson, Caitlin Davies, Stephen M. Lawrie

**Affiliations:** Division of Psychiatry, University of Edinburgh, Royal Edinburgh Hospital, Edinburgh, UK

**Keywords:** Personalised, precision, stratified, medicine, psychosis, patient-centred

## Abstract

**Background:**

Single patient or ‘*n*-of-1’ trials are a pragmatic method to achieve optimal, evidence-based treatments for individual patients. Such trials could be particularly valuable in chronic, heterogeneous, difficult to treat illnesses such as schizophrenia.

**Aims:**

To identify how often, and in what way, *n*-of-1 trials have been used in schizophrenia.

**Method:**

We performed a systematic search in the major electronic databases for studies adopting *n*-of-1 methodology in schizophrenia, published in English from the start of records until the end of January 2017.

**Results:**

We identified six studies meeting inclusion criteria. There was wide variability in study methodology and analysis. Each trial reported positive outcomes for their respective intervention, but all studies were at high risk of bias.

**Conclusions:**

In conclusion, *n*-of-1 trials are currently underutilised in schizophrenia. Existing trials suggest the method is well tolerated and potentially effective in achieving optimal treatments for patients, but more standardised methods of design, execution and analysis are required in future trials.

**Declaration of interest:**

S.M.L. has received grants and personal fees from Janssen, and personal fees from Otsuka and Sunovion, in the past 3 years, outside the submitted work.

## Key concepts in n-of-1 trials

*n*-of-1 trials are arguably the future of evidence-based care in schizophrenia. An *n*-of-1 trial is simply a prospective crossover study of a single patient exposed to different treatment conditions. For example, repetitively comparing a treatment (A) against no treatment (B) (AB, BA) or comparing treatment A against no treatment, against a second treatment C (AB, AC). *n*-of-1 trials are particularly useful for chronic conditions that are relatively stable over time and where substantial clinical uncertainty exists over the best treatment – such as choice of antipsychotic or other treatment selection in schizophrenia.

*n*-of-1 trials are very similar to normal ‘trial and error’ clinical practice but with additional measures to reduce bias. Key measures include: balanced treatment *v.* comparison sequence assignment (which may be aided by randomisation if a large number of treatment blocks are used), blinding (to the extent to which this is possible), and systematic outcome measurements.^1^ For psychotropic medications where onset and cessation of effect are slow, building in appropriate run-in and wash-out periods to the design is also important.

## Relevance to schizophrenia

*n*-of-1 trials have existed for decades yet are only now becoming increasingly important in psychiatry and other branches of medicine for three main reasons. First, the improved understanding of the genetic determinants of disease means that personalised treatment based on a patient's particular genetic risk factors is now a reality for some (for example in cancer^2^ and epilepsy^3^) and likely to be available to all in years to come.^4^ Conceivably thousands of individualised treatments may be indicated to treat highly penetrant ultra-rare or *de novo* genetic variants associated with a large proportion of severe intellectual disability,^5^ autism^6^ and, to a lesser extent, schizophrenia.^7^ The discovery of auto-antibodies against neurotransmitter receptors underlying a subset of psychotic disorders that previously could have been diagnosed as schizophrenia also emphasises that schizophrenia is not a homogeneous entity.^8^ The uncovering of the likely heterogeneity of mechanisms underlying psychiatric disorders such as schizophrenia means a robust method of assessing response to treatment is required at the individual level, in addition to the group.

Second, *n*-of-1 trials are the epitome of patient-centred care. They can be used to help those patients whom randomised controlled trials fail: those with comorbid physical, mental or substance use problems, those requiring concurrent medication and those at the extremes of age. Many of these limitations of generalisability apply in medication trials in schizophrenia. *n*-of-1 trials remove the altruistic component of trial participation and give benefit direct to the individual, which is particularly important for vulnerable groups. Further, *n*-of-1 trials are easily adaptable to patient preference in terms of interventions trialled and outcome measures assessed. This is important because patient engagement improves outcomes in chronic illness^9^ and participation in *n*-of-1 trials for conditions such as attention-deficit hyperactivity disorder can increase empowerment and feelings of control.^10^

Finally, *n*-of-1 trials are coming of age as technology to facilitate the systematic assessment of health outcomes is increasingly affordable and available. Regular assessment of predefined measures of response and harm is a key component of an *n*-of-1 trial. Historically, this has been a resource-intensive process requiring frequent clinician–participant interactions, but the availability of smart phones and ancillary devices (able to record measures such as heart rate, vocal stress and electroencephalograms) means that regular assessment of both physiological and subjective parameters is now much more readily available. Such technology has been harnessed by a recent *n*-of-1 trial series assessing the impact of statins on muscle pain^11^ and self-monitoring mobile phone applications are already showing feasibility and benefit in mental health disorders,^12^ including psychotic conditions.^13^

People with schizophrenia are therefore a key group who are likely to benefit from *n*-of-1 trials. However, the number and value of existing *n*-of-1 trials in this patient group is currently unknown. We thus systematically reviewed the available literature on *n*-of-1 trials in patients with schizophrenia to establish how commonly they are used, the approach to their application, the acceptability of the method to the patient group, and the strengths and weaknesses of existing trials. Our findings aim to inform and improve *n*-of-1 trials in schizophrenia in the future.

## Method

### Search strategy and selection criteria

We searched MEDLINE (Ovid interface, Ovid MEDLINE in-process and other non-indexed citations and Ovid MEDLINE 1946 onwards), Embase (Ovid interface, 1980 onwards), Cochrane Library (Wiley online platform), Web of Science (core collection) and PsycINFO (Ovid interface 1987 to current) for relevant articles indexed as of 2 February 2017. The following search terms were used (“N-of-1” OR “single patient trial” OR (“individual” OR “single” OR “within”) AND (“patient” or “participant” or “subject”)) OR (“case stud*” AND “experiment”)) AND (“schizophrenia” OR “Psychotic” OR “neuroleptic malignant syndrome” OR “dyskinesia”), and their synonyms, as described in Supplementary Appendix 1 available at https://doi.org/10.1192/bjp.2018.70. Papers citing influential early ‘*n*-of-1’ publications^14^^–^^16^ were also screened for their own eligibility.

All titles and abstracts yielded by the searches were screened by A.J.S., with a random sample of 10% screened by C.D. and discrepancies resolved by discussion. The full texts were screened by A.J.S. We included any peer-reviewed studies that exploited an *n*-of-1 study design as a treatment trial for an individual patient with schizophrenia. We defined *n*-of-1 studies as those that adhered to the definition described in the Consolidated Standards of Reporting Trials (CONSORT) extension for reporting N-of-1 Trials (CENT) 2015 – a single participant trial, using a repeated series of treatment challenge and withdrawal, where one cycle (A) is the intervention being investigated and the other (B) is either a comparative treatment, a control or no intervention.^17^ Studies that provided insufficient trial detail or were not published in English were excluded.

Data were extracted independently by A.J.S. and K.F.M.M. Data extracted from each study were: first author, year of publication, country of study, duration of study, patient characteristics (age, sex and diagnosis), trial design, intervention(s) used, comparator used, blinding measures, primary outcome measures, method of analysis, results and other outcomes. We adapted the CENT 2015 checklist^17^ to assess how well the studies were reported (Supplementary Table 1). We also assessed the quality of studies using the Cochrane risk of bias criteria, commonly used when assessing clinical trials.^18^ See Supplementary Table 2 for the PRISMA checklist for this review. We did not register a review protocol. Ethics committee approval was not required.

## Results

### Study characteristics

**Fig. 1:**
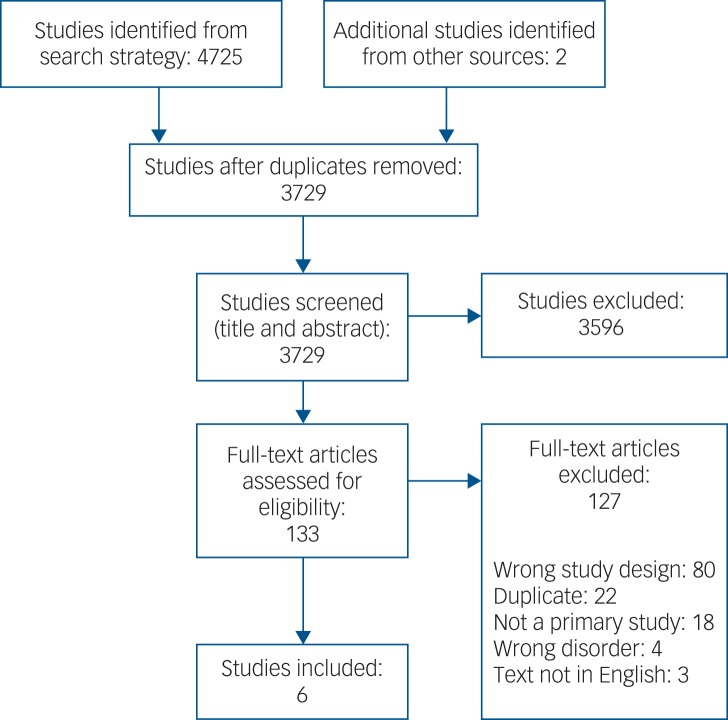
Study selection.

We assessed 4727 citations, of which six studies met inclusion criteria (Fig. 1). The included studies are summarised in Table 1.

**Table 1:** Study characteristics with key patient data

	Patient characteristics	Trial design	Duration	Intervention used	Comparator used	Blinding	Main outcome Measure(s)	Analysis	Main Result(s)
Alford (1986)^19^	22-year-old man with chronic paranoid schizophrenia (DSM III) receiving fluphenazine decanoate	ABAB	Not reported	Cognitive intervention (Social reinforcement-assisted)	Placebo (general conversation)	Patient and treating physician not Blinded. Blinded secondary outcome assessors	Self-reports of frequency and strength of delusional belief, and frequency of Thorazine (when required) use	Qualitative and graphical	Decreasing frequency and strength of belief in delusion ideation during treatment phases. Decreased administration of Thorazine (when required)
Blanco-Lopez *et al* (2016)^24^	18-year-old woman with schizophrenia receiving clozapine	BAB	8 weeks	Transcranial magnetic stimulation of left temporoparietal cortex	Treatment as usual	None reported	Unclear but appeared to be self-report of duration of time without auditory hallucinations and depressive symptoms. Also clinical global impression	Qualitative	Decreasing frequency of auditory hallucinations. Improved mood. Improved clinical impression
Done *et al* (1986)^23^	42-year-old man with chronic schizophrenia receiving flupentixol decanoate 150 mg weekly. Target symptom auditory hallucinations	ABAC	5 months	Right ear plug	No ear plug (B) and left ear plug only (C)	None reported	Self-report of frequency, volume and disturbing content of hallucinations	Non-parametric statistics	Right ear plug reduced the frequency and volume of hallucinations compared with left ear plug or no plug
Gorczynski *et al* (2014)^22^	4 patients, all with chronic schizophrenia: a 29-year-old man receiving clozapine; a 28-year-old man receiving loxapine; a 25-year-old woman receiving clozapine, and ‘weight reduction medication’; a 36-year-old woman receiving clozapine	ABA	10 weeks	Exercise counselling	Treatment as usual	None reported	Self-report of stage of change, self-efficacy and benefits and barriers. Objective assessment of exercise via accelerometer	Parametric statistics, graphical and tabular	No effect on objective assessment of exercise. Self-report indicated progress through stages of change and minor increases in self-efficacy
Kay & Opler (1985)^21^	55-year-old woman with chronic paranoid schizophrenia (DSM III) receiving haloperidol 15 mg	Reversal design (ABA)	27 weeks	L-dopa (Sinemet) (combined with haloperidol)	Placebo (combined with haloperidol)	Double-blind	Brief Psychiatric Rating Scale, Psychopathology Rating Schedule, Span of Attention Test	Non-parametric statistics	Significant improvement in negative symptoms. No change in positive symptoms
MacEwan *et al* (2001)^20^	36-year-old man with schizophrenia-paranoid type (in partial remission) receiving risperidone 2 mg	ABAB	30 weeks	Donepezil (10 mg daily)	Treatment as usual	None	Battery of 9 standardised cognitive tests. Self-report of functioning	Simple tabulation and qualitative summary	Increased verbal fluency and subjective concentration

Five studies reported on a single patient: three men and two women. One study reported an *n*-of-1 series involving four participants (two men and two women). All participants had received a diagnosis of schizophrenia and were stabilised on antipsychotic medication. Patient ages ranged from 18 to 55 years old, and each had a prolonged history of psychosis and psychiatric intervention, ranging from 2 to 22 years. As expected, each patient's presenting problem, the rationale for employing an *n*-of-1 approach and the discrete objectives for using each intervention differed greatly between studies.

Study length ranged from 8 to 30 weeks, although one study failed to report duration. Trial designs were diverse with minimal replication: two of the studies adopted an ABAB crossover design,^19^^,^^20^ two an ABA reversal design,^21^^,^^22^ one an ABAC design^23^ and one a BAB design.^24^ The nature of interventions trialled was also wide ranging: two studies investigated pharmacological interventions (L-dopa^21^ and donepezil^20^), two a psychological intervention (‘social reinforcement-assisted cognitive intervention’^19^ and exercise counselling^22^), and two a physical intervention (ear plug and transcranial magnetic stimulation). Comparison phases were varied, comprising a mixture of placebos (inactive tablet,^21^ ear plug in opposite ear,^23^ normal conversation^19^) and treatment as usual.^20^^,^^22^^,^^24^

Two studies reported blinding measures, with one blinding both the patient and the evaluating physician,^21^ and the other only blinding the secondary outcome assessors.^19^ Of the other four studies, one discloses that no blinding occurred^20^ and the others fail to declare whether or not any measures of blinding were applied.^22^^–^^24^

Outcome measures largely comprised assessments of psychological functioning; three papers used standardised assessments and the other three tailored their outcome measures to the individual patient and intervention in question (Table 1). Four of the studies employed both subjective and objective outcome measures.^19^^–^^22^ In one case the objective measure (frequency of exercise via an accelerometer) showed no benefit from the intervention, in contrast to some of the self-report measures.^22^ Frequency of collection of outcome measures varied considerably. The description of results and analytical measures were largely underreported or underemployed: three papers offered graphical and tabulated data and a qualitative summary^19^^,^^20^^,^^22^ but did not report comprehensive raw data scores. Statistical tests were employed by three studies but lacked both full reporting of the tests and the raw data used.^21^^–^^23^ One study gave a selective sample of raw data only.^24^

Five of the studies had positive outcomes for the intervention under investigation; two papers that reported statistical analysis showed some statistically significant benefit of the experimental treatment in question^21^^,^^23^ whereas the studies which described outcomes qualitatively described a trend of improvement. The study reporting on exercise counselling had mixed findings: it did not find an objective benefit of the intervention but did find some improvement in self-report measures.^22^ One paper reported that their patient continued on the treatment that was being assessed^20^ and one study found that the outcome remained highly positive several months later without any further intervention required.^24^ Two other studies reported qualified positive outcomes of follow-up assessments^19^^,^^23^ but did not indicate if the treatment under investigation was continued.

### Quality assessment

Currently, there are no standardised measures for quality assessment in *n*-of-1 trials so we assessed studies using a standard measure of risk of bias often used in assessments of randomised controlled trials^18^^,^^25^ (Table 2). Overall, the trials were assessed as at high risk of bias, with reduced quality because of insufficient reporting of trial detail, insufficient blinding and insufficient numbers of replications of treatment pairs, without opportunity for randomisation or counterbalancing. We also used the CENT 2015 checklist to provide a measure of the completeness and clarity of the reporting of the included studies, which again highlighted gaps in reporting (see Supplementary Table 1).

**Table 2:** Cochrane risk of bias table, modified for *n*-of-1 trials

Bias reduction measure	Application to *n*-of-1	Alford (1986)^19^	Blanco-Lopez *et al* (2016)^24^	Done *et al* (1986)^23^	Gorczynski *et al* (2014)^22^	Kay & Opler (1985)^21^	MacEwan *et al* (2001)^20^
Random sequence generation	Counterbalancing more appropriate than randomisation if small number of blocks	X	X	X	X	X	X
Allocation concealment	Less important than in randomised controlled trial as crossover design: both treatments will be received over time	X	X	X	X	X	X
Blinding (participants and personnel)	May be harder in small-scale, naturalistic trials. May be less relevant as non-specific effects still worthwhile	X	X	X	X	✓	X
Blinding (outcome assessors)	Particularly difficult if patient is collecting data on themselves	?	X	X	X	✓	X
Completeness of outcome data	for *n*-of-1 trials, participant drop-out not relevant, but missing data for main outcomes still relevant	X	X	X	?	X	X
All outcomes reported		X	?	X	✓	X	X
Other sources of bias minimised	for *n*-of-1 trials, key source of bias is carry-over of treatments and variation of outcome by time independently of treatment. This can be minimised by counterbalanced treatment orders and by multiple blocks (replication)	X	X	X	X	X	X

X, high risk; ?, unclear risk; ✓, low risk.

## Discussion

### Main findings

The most prominent finding of this review is the paucity of *n*-of-1 trials in the current schizophrenia literature. We found only six studies that corresponded to our inclusion criteria, indicating that the increasing recognition of *n*-of-1 trials and declarations of interest in personalised psychiatry has not yet translated to research in schizophrenia. Comparison of the key study characteristics reveals the variability in methodological and interventional approaches adopted in such trials: we found one study investigating whether or not an ear plug could reduce auditory hallucinations, one assessing whether transcranial magnetic stimulation could reduce auditory hallucinations, one testing if L-dopa could alleviate negative symptoms, one assessing adjunctive use of donepezil, one using a cognitive intervention to alter delusional beliefs and one assessing the benefits of exercise counselling on activity. The studies encompassed the spectrum of psychiatric treatment modalities, which is promising, indicating this form of trial is viable in the investigation of the range of interventions that are often considered in the complex management of schizophrenia and other psychiatric disorders. None of the trials reported any adverse effects, or any issues with patient adherence or acceptability, and they mainly reported positive outcomes, suggesting that such trials are both feasible and potentially valuable in patients with schizophrenia.

### Risk of bias

The studies were, however, all considered to be at high risk of bias, largely because of a lack of clarity and depth in their reporting and because they did not include key design features such as blinding and counterbalanced treatment conditions. Our results are similar to comprehensive reviews looking at *n*-of-1 trials across the medical literature, suggesting these issues need addressing throughout medicine.^26^^,^^27^ All but one of the studies were published before trial reporting guidelines,^17^ and the availability of these should improve future *n*-of-1 trial reporting.

Blinding of the treatment provider and to a lesser extent the patient will always be more difficult in studies providing non-pharmacological interventions, whether the study is a randomised control trial^28^ or an *n*-of-1 trial. However, blinding of outcome assessors remains possible, and this also was lacking in the majority of studies.

Additional areas that all of the studies here could have improved on would have been to reduce the risk of bias because of natural variation over time by having more replication cycles (the largest number of cycles was two) and by counterbalancing the treatment and comparison arms (e.g. AB, BA rather than AB, AB). Longer trial durations would also have reduced the risk of measuring placebo and Hawthorne (halo) effects (change in behaviour simply because of the attention received through being a research participant).^29^ However, one trial found complete resolution of symptoms following the second round of treatment, suggesting that further treatment cycles would be a waste of resource and even potentially harmful. Further, psychological interventions by their nature are designed to have effects with duration beyond the period of treatment, limiting the potential for multiple within-patient replications. The diversity of even the small number of *n*-of-1 trials considered here highlights the difficulty in dictating quality standards that balance rigour with feasibility.

### Use of patient-reported outcomes

Another challenge for future *n*-of-1 trials is to harness the potential power of patients reporting their own outcomes (e.g. via smart phones) while also ensuring objective assessments. Of the four studies that reported both subjective and objective measures, the findings conflicted in one. Overreliance on subjective assessment can occur in group trials as well as *n*-of-1 trials, but *n*-of-1 trials are made particularly vulnerable by their highly patient-centred, sometimes bespoke and often low-resource nature. Patient satisfaction with an intervention is important, but it is not the same as effectiveness.

### Limitations

We designed a comprehensive search strategy to ensure we captured all examples of *n*-of-1 treatment trials in patients with schizophrenia that conformed to our definition; however, it is possible we overlooked some relevant examples. One reason for this, and an area of *n*-of-1 trials that needs work, is the lack of consensus in the nomenclature; there is no widely accepted, single definition of what constitutes an *n*-of-1 methodology, with various opinions on a number of fundamental aspects including the necessity for blinding and randomisation and the requisite number of treatment cycles.^1^^,^^30^ This variation reflects the adaptability and diversity of *n*-of-1 methodology, with different approaches and durations required for trials using pharmacological *v.* psychological interventions, interventions with a substantial *v.* a subtle impact, conditions with rapid *v.* slow symptom responses and medications with short *v.* long speeds of onset and discontinuation.

Furthermore, none of the papers included in this review explicitly identify themselves as an *n*-of-1 trial; instead they designate themselves as ‘a single-subject experimental study’ (two studies), ‘a single-case experimental analysis’, a ‘single patient study’ and ‘an experimental design case study’, so although they do conform to the CENT 2015 definition of *n*-of-1 trials this is perhaps more by accident than by design. This means it can be challenging to pick out such studies in the literature. We will also have missed any studies published in a language other than English, and any non-peer-reviewed studies in the ‘grey literature’.

It is also highly probable that not all *n*-of-1 trials that are executed are published because of a lack of awareness of their potential broader clinical value as, by design, they result in findings that defy simple generalisation, meaning motivation to publish results may not be high. This is particularly likely to be the case with negative findings, potentially placing *n*-of-1 trials at higher risk of publication bias than trial designs in which pre-registration is the expectation. Although the purpose of *n*-of-1 trials is to objectively determine the optimal intervention for an individual using data-driven criteria, generalisability and population-level clinical value can be achieved by combining data from series of comparable *n*-of-1 trials using meta-analytic statistical approaches, predominantly Bayesian.^31^^,^^32^ Similar statistical approaches have also been developed to interpret data from related trial methodologies such as sequential multiple assignment randomised trials (SMART)^33^ (e.g. the CATIE study^34^), which could be viewed as *n*-of-1 trials with a predefined sequence of treatment options and decision points, and individual participant data meta-analysis.^35^ *n*-of-1 trials therefore represent a method flexible enough for individual treatment optimisation that could also be standardised for wider research into subsets of the population. Awareness of this value should encourage their use, and may reassure pharmaceutical companies and regulatory bodies that *n*-of-1 trials can provide evidence strong enough to support marketing and licensing decisions for specific groups.

### Future directions

Despite the many potential benefits of *n*-of-1 trials, there has not yet been a rise in such studies in the field of schizophrenia. Our review suggests this is not because of a lack of feasibility or utility but perhaps a lack of awareness of their value and the availability of a formalised approach to their design and conduct. Resources are now available for researchers to effectively plan, execute and analyse such trials, allowing them to attain valuable results for their patients, and potentially the wider population of those with a diagnosis of schizophrenia. We hope that the next decade will see a blossoming of high quality *n*-of-1 trials of various therapeutic strategies in the management of schizophrenia and related conditions.

## Supplementary material

For supplementary material accompanying this paper visit https://doi.org/10.1192/bjp.2018.71. click here to view supplementary material
