# Application of *n*-of-1 treatment trials in schizophrenia: systematic review – ERRATUM

**DOI:** 10.1192/bjp.2018.117

**Published:** 2018-08

**Authors:** Katie F. M. Marwick, Anna J. Stevenson, Caitlin Davies, Stephen M. Lawrie

## Abstract

This notice describes a correction to the above mentioned paper.

The first two authors of this paper, Katie F. M. Marwick and Anna J. Stevenson, contributed equally to this paper and are joint first authors. The publisher apologises for this omission on the original publication of the paper.
