# Supplementary material for: Application of n-of-1 treatment trials in schizophrenia: systematic review
Source: Br J Psychiatry. 2018 Jul;213(1):398–403. doi: 10.1192/bjp.2018.71 (PMC6025794; doi:10.1192/bjp.2018.71)
Supplement: Supplementary file 1 [file S0007125018000715sup.zip › S0007125018000715sup001.docx]

# Supplementary Information

## Supplementary Table 1 CENT 2015 checklist: CONSORT 2010 checklist items with modifications or additions for individual or series of N-of-1 trials.

✔: reported. x: not reported

| **Section/topic** | **CONSORT 2010** | | **CENT 2015** |  | **Study** | | | | | |
| --- | --- | --- | --- | --- | --- | --- | --- | --- | --- | --- |
|  | **No.** | **Item** | **No.** | **Item** | **Alford 1986** | **Blanco-Lopez 2016** | **Done 1986** | **Gorcyznski 2014** | **Kay 1985** | **MacEwan 2001** |
| **Title and abstract** | 1a | Identification as a randomised trial in the title | 1a | Identify as an “N-of-1 trial” in the title | x | x | x | x | x | x |
|  |  |  |  |  |  |  |  |  |  |  |
|  | 1b | Structured summary of trial design, methods, results, and conclusions (for specific guidance see CONSORT for abstracts) | 1b |  | ✔ | x | x | ✔ | ✔ | ✔ |
| **Introduction** | 2a | Scientific background and explanation of rationale | 2a.1 |  | ✔ | ✔ | ✔ | ✔ | ✔ | ✔ |
| Background and objectives |  |  |  |  |  |  |  |  |  |  |
|  |  |  | 2a.2 | Rationale for using N-of-1 approach | ✔ | x | ✔ | ✔ | ✔ | ✔ |
|  | 2b | Specific objectives or hypotheses | 2b |  | ✔ | ✔ | ✔ | ✔ | ✔ | ✔ |
| **Methods** | 3a | Description of trial design (such as parallel, factorial) including allocation ratio | 3a | Describe trial design, planned number of periods, and duration of each period (including run-in and wash out, if applicable) | ✔ | ✔ | ✔ | ✔ | ✔ | ✔ |
| Trial design |  |  |  |  |  |  |  |  |  |  |
|  | 3b | Important changes to methods after trial start (such as eligibility criteria), with reasons | 3b |  | ✔ | x | ✔ | x | x | x |
| Participant(s) | 4a | Eligibility criteria for participants | 4a | Diagnosis or disorder, diagnostic criteria, comorbid conditions, and concurrent therapies | ✔ | ✔ | ✔ | ✔ | ✔ | ✔ |
|  | 4b | Settings and locations where the data were collected | 4b |  | ✔ | x | ✔ | ✔ | x | x |
|  |  |  | 4c | Whether the trial(s) represents a research study and if so, whether institutional ethics approval was obtained | x | ✔ | x | ✔ | x | x |
| Interventions | 5 | The interventions for each group with sufficient details to allow replication, including how and when they were actually administered | 5 | The interventions for each period with sufficient details to allow replication, including how and when they were actually administered | ✔ | ✔ | ✔ | ✔ | ✔ | ✔ |
| Outcomes | 6a | Completely defined pre-specified primary and secondary outcome measures, including how and when they were assessed | 6a.1 |  | ✔ | x | ✔ | ✔ | ✔ | ✔ |
|  |  |  | 6a.2 | Description and measurement properties (validity and reliability) of outcome assessment tools | ✔ | x | x | ✔ | ✔ | ✔ |
|  | 6b | Any changes to trial outcomes after the trial commenced, with reasons | 6b |  | x | x | ✔ | x | x | x |
| Randomisation: | 8a | Method used to generate the random allocation sequence | 8a | Whether the order of treatment periods was randomised, with rationale, and method used to generate allocation sequence | x | x | x | x | x | x |
| Sequence generation |  |  |  |  |  |  |  |  |  |  |
|  | 8b | Type of randomisation; details of any restriction (such as blocking and block size) | 8b | When applicable, type of randomisation; details of any restrictions (such as pairs, blocking) | - | - | - | - | - | - |
|  |  |  | 8c | Full, intended sequence of periods | ✔ | ✔ | ✔ | ✔ | ✔ | ✔ |
| Allocation concealment mechanism | 9 | Mechanism used to implement the random allocation sequence (such as sequentially numbered containers), describing any steps taken to conceal the sequence until interventions were assigned | 9 |  | x | x | x | x | x | x |
| Implementation | 10 | Who generated the random allocation sequence, who enrolled participants, and who assigned participants to interventions | 10 |  | x | x | x | x | x | x |
| Blinding | 11a | If done, who was blinded after assignment to interventions (for example, participants, care providers, those assessing outcomes) and how | 11a |  | ✔ | x | ✔ | x | ✔ | ✔ |
|  | 11b | If relevant, description of the similarity of interventions | 11b |  | - | - | - | - | x | - |
| Statistical methods | 12a | Statistical methods used to compare groups for primary and secondary outcomes | 12a | Methods used to summarize data and compare interventions for primary and secondary outcomes | x | x | ✔ | ✔ | ✔ | x |
|  | 12b | Methods for additional analyses, such as subgroup analyses and adjusted analyses | 12b |  | ¯ | - | ¯ | - | ✔ | ¯ |
|  |  |  | 12c | Statistical methods used to account for carryover effect, period effects, and intra-subject correlation | x | x | x | x | x | x |
| **Results** | 13a | For each group, the numbers of participants who were randomly assigned, received intended treatment, and were analysed for the primary outcome | 13a.1 | Number and sequence of periods completed, and any changes from original plan with reasons | ✔ | ✔ | ✔ | ✔ | ✔ | ✔ |
| Participant flow |  |  |  |  |  |  |  |  |  |  |
|  | 13b | For each group, losses and exclusions after randomisation, together with reasons | 13b |  | ¯ | - | ¯ | - | ¯ | ¯ |
| Recruitment | 14a | Dates defining the periods of recruitment and follow-up | 14a |  | x | x | x | x | x | x |
|  | 14b | Why the trial ended or was stopped | 14b | Whether any periods were stopped early and/or whether trial was stopped early, with reason(s). | x | x | x | x | x | x |
| Baseline data | 15 | A table showing baseline demographic and clinical characteristics for each group | 15 |  | x | x | x | ✔ | x | x |
| Numbers analysed | 16 | For each group, number of participants (denominator) included in each analysis and whether the analysis was by original assigned groups | 16 | For each intervention, number of periods analysed. | ✔ | ✔ | ✔ | ✔ | ✔ | x |
| Outcomes and estimation | 17a | For each primary and secondary outcome, results for each group, and the estimated effect size and its precision (such as 95% confidence interval) | 17a.1 | For each primary and secondary outcome, results for each period; an accompanying figure displaying the trial data is recommended. | ✔ | ? | x | ✔ | x | x |
|  |  |  | 17a.2 | For each primary and secondary outcome, the estimated effect size and its precision (such as 95% confidence interval) | x | x | x | x | x | x |
|  | 17b | For binary outcomes, presentation of both absolute and relative effect sizes is recommended | 17b |  | x | x | x | x | x | x |
| Ancillary analyses | 18 | Results of any other analyses performed, including subgroup analyses and adjusted analyses, distinguishing pre-specified from exploratory | 18 | Results of any other analyses performed, including assessment of carryover effects, period effects, intra-subject correlation | x | x | ✔ | x | ✔ | x |
| Harms | 19 | All important harms or unintended effects in each group (for specific guidance see CONSORT for harms) | 19 | All harms or unintended effects for each intervention | x | x | x | x | x | x |
| **Discussion** | 20 | Trial limitations, addressing sources of potential bias, imprecision, and, if relevant, multiplicity of analyses | 20 |  | ✔ | x | x | ✔ | ✔ | ✔ |
| Limitations |  |  |  |  |  |  |  |  |  |  |
| Generalisability | 21 | Generalisability (external validity, applicability) of the trial findings | 21 |  | ✔ | x | ✔ | x | ✔ | ✔ |
| Interpretation | 22 | Interpretation consistent with results, balancing benefits and harms, and considering other relevant evidence | 22 |  | ✔ | ✔ | ✔ | ✔ | ✔ | ✔ |
| **Other information** | 23 | Registration number and name of trial registry | 23 |  | x | x | x | x | x | x |
| Registration |  |  |  |  |  |  |  |  |  |  |
| Protocol | 24 | Where the full trial protocol can be accessed, if available | 24 |  | x | x | x | x | x | x |
| Funding | 25 | Sources of funding and other support (such as supply of drugs), role of funders | 25 |  | x | x | x | x | x | x |

## *Appendix 1.* Search Strategy

**MEDLINE search strategy (Ovid MEDLINE(R) In-Process & Other Non-Indexed Citations and Ovid MEDLINE(R) 1946 to Present)**

1. N-of-1.tw, mp
2. "n of 1".tw, mp
3. ((individual or single or within) adj (patient* or participant* or subject*)),tw
4. (case stud* adj2 experiment*).tw
5. “single patient trial”.tw
6. “n=1”.mp
7. or/1-6
8. Exp schizophrenia
9. Exp paranoid disorders
10. Schizo&.mp
11. Hebephrenic*.mp
12. Oligophreni*.mp
13. Psychotic*.mp
14. Psychosis.mp
15. Psychoses.mp
16. ((chronic* or sever*) adj2 mental* adj2 (ill* or disorder*)).mp
17. Exp dyskinesia, drug-induced/
18. Exp psychomotor agitation/
19. Exp neuroleptic malignant syndrome/
20. Exp "diagnosis, dual (psychiatry)"/
21. (tardive* adj dyskine*).mp
22. Akathisi*.mp
23. Acathisi*.mp
24. (neuroleptic* and (malignant adj2 syndrome)).mp
25. (neuroleptic* and (movement and disorder*)).mp
26. Neuroleptic-induc*.mp
27. Or/ 8-26
28. 7 and 27

**EMBASE search strategy – (Embase 1980 to Present)**

1. n-of-1..mp, tw
2. "n of 1".mp, tw
3. ((individual or single or within) adj (patient* or participant* or subject*) adj1 (trial or experiment*)).tw
4. (case stud* adj2 experiment*).tw
5. "single patient trial*".mp
6. 1 or 2 or 3 or 5
7. (schizo* or psychotic* or psychosis or psychoses).mp
8. exp schizophrenia/
9. exp psychosis/
10. mental patient/
11. (tardiv* adj dyskine*).mp
12. neuroleptic agent/
13. (neuroleptic* and (malignant adj2 syndrome)).mp
14. tardive dyskinesia/
15. akathisia/
16. exp neuroleptic malignant syndrome/
17. (neuroleptic* and movement and disorder*).mp
18. 7 or 8 or 9 or 10 or 11 or 12 or 13 or 14 or 15 or 17
19. 7 and 18

**PsycINFO search strategy – PsycINFO 1987 to current**

1. n-of-1.tw, mp
2. "n of 1".tw, mp
3. ((individual or single or within) adj (patient* or participant* or subject*)).tw
4. (case stud* adj2 experiment*).mp
5. “single patient trial”.mp
6. or/1-4
7. schizo*.mp
8. oligophreni*.mp
9. psychotic*.mp
10. psychosis.mp
11. psychoses.mp
12. exp psychosis/
13. exp schizophrenia/
14. exp schizoaffective disorder/
15. (tardiv* adj dyskine*).mp
16. akathisi*.mp
17. acathisi*.mp
18. (neuroleptic* and (malignant adj2 syndrome)).mp
19. (neuroleptic* and (movement and disorder*)).mp
20. exp neuroleptic malignant syndrome/
21. exp dyskinesia/
22. exp akathisia
23. neuroleptic-induc*.mp
24. or/ 6-23

**Web of Science search strategy – (Web of Science Core Collection)**

1. Topic =(“n-of-1”)
2. Topic =(“n of 1”)
3. Topic =(“single” or “individual” or “within”) near/1 (“patient$” or “participant$” or “subject$”)
4. (#1 or #2 or #3)
5. Topic =(schizo$)
6. Topic =(psychosis$)
7. Topic =(psychotic$)
8. Topic =(psychoses$)
9. Topic =(oligophreni$)
10. Topic =(hebephreni$)
11. Topic =(tardiv$ near dyskine$)
12. Topic =(akathisi$)
13. Topic =(acathisi$)
14. Topic =(“neuroleptic malignant syndrome$”)
15. Topic =(“severe mental illness$”)
16. Topic =(“chronic mental illness$”)
17. Topic =(“chronic mental disorder$”)
18. Topic =(“severe mental disorder$”)
19. Topic =(neuroleptic-induc$)
20. (#5 or #6 or #7 or #8 or #9 or #10 or #11 or #12 or #13 or #14 or #15 or #16 or #17 or #18 or #19)
21. (#4 and #20)

**Cochrane Library search strategy – (Cochrane Reviews (Reviews only), Other Reviews, Trials, Methods Studies, Technology Assessments, Economic Evaluations and Cochrane Groups)**

1. "n-of-1"
2. ((individual or single) next (patient* or participant* or subject*))
3. (#1 or #2)
4. Schizophrenia
5. "paranoid disorders"
6. schizo*
7. hebephreni*
8. oligophreni*
9. psychotic*
10. psychosis
11. psychoses
12. (tardiv* next dyskine*)
13. "neuroleptic malignant syndrome"
14. acathisi*
15. akathisi*
16. (#4 or #5 or #6 or #7 or #8 or #9 or #10 or #11 or #12 or #13 or #14 or #15)
17. (#3 and #16)
